# Therapist-Assisted Internet-Based Cognitive Behavioral Therapy Versus Progressive Relaxation in Obsessive-Compulsive Disorder: Randomized Controlled Trial

**DOI:** 10.2196/jmir.9566

**Published:** 2018-08-08

**Authors:** Michael Kyrios, Claire Ahern, Daniel B Fassnacht, Maja Nedeljkovic, Richard Moulding, Denny Meyer

**Affiliations:** ^1^ College of Education, Psychology & Social Work Flinders University Adelaide Australia; ^2^ Research School of Psychology The Australian National University Canberra Australia; ^3^ Department of Psychological Sciences School of Health Sciences Swinburne University of Technology Melbourne Australia; ^4^ School of Psychology Faculty of Health Deakin University Geelong Australia; ^5^ Centre for Drug Use, Addictive and Anti-Social Behaviour Research Deakin University Geelong Australia; ^6^ Department of Statistics Data Science and Epidemiology School of Health Sciences Swinburne University of Technology Melbourne Australia

**Keywords:** obsessive-compulsive disorder, mental health, cognitive behavioral therapy, CBT, online intervention

## Abstract

**Background:**

Obsessive-compulsive disorder (OCD) is a highly disabling psychological disorder with a chronic course if left untreated. Cognitive behavioral therapy (CBT) has been shown to be an effective treatment, but access to face-to-face CBT is not always possible. Internet-based CBT (iCBT) has become an increasingly viable option. However, no study has compared iCBT to an analogous control condition using a randomized controlled trial (RCT).

**Objective:**

A 2-armed RCT was used to compare a therapist-assisted 12-module iCBT to an analogous active attention control condition (therapist-assisted internet-based standard progressive relaxation training, iPRT) in adult OCD. This paper reports pre-post findings for OCD symptom severity.

**Method:**

In total, 179 participants (117 females, 65.7%) were randomized (stratified by gender) into iCBT or iPRT. The iCBT intervention included psychoeducation, mood and behavioral management, exposure and response prevention (ERP), cognitive therapy, and relapse prevention; the iPRT intervention included psychoeducation and relaxation techniques as a way of managing OCD-related anxiety but did not incorporate ERP or other CBT elements. Both treatments included audiovisual content, case stories, demonstrations of techniques, downloadable audio content and worksheets, and expert commentary. All participants received 1 weekly email, with a maximum 15-minute preparation time per client from a remote therapist trained in e-therapy. Emails aimed to monitor progress, provide support and encouragement, and assist in individualizing the treatment. Participants were assessed for baseline and posttreatment OCD severity with the telephone-administered clinician-rated Yale-Brown Obsessive-Compulsive Scale and other measures by assessors who were blinded to treatment allocation.

**Results:**

No pretreatment differences were found between the 2 conditions. Intention-to-treat analysis revealed significant pre-post improvements in OCD symptom severity for both conditions (*P*<.001). However, relative to iPRT, iCBT showed significantly greater symptom severity improvement (*P*=.001); Cohen *d* for iCBT was 1.05 (95% CI 0.72-1.37), whereas for iPRT it was 0.48 (95% CI 0.22-0.73). The iCBT condition was superior in regard to reliable improvement (25/51, 49% vs 16/55, 29%; *P*=.04) and clinically significant pre-post-treatment changes (17/51, 33% vs 6/55, 11%; *P*=.005). Those undertaking iCBT post completion of iPRT showed further significant symptom amelioration (*P*<.001), although the sequential treatment was no more efficacious than iCBT alone (*P*=.63).

**Conclusion:**

This study is the first to compare a therapist-assisted iCBT program for OCD to an analogous active attention control condition using iPRT. Our findings demonstrate the large magnitude effect of iCBT for OCD; interestingly, iPRT was also moderately efficacious, albeit significantly less so than the iCBT intervention. The findings are compared to previous internet-based and face-to-face CBT treatment programs for OCD. Future directions for technology-enhanced programs for the treatment of OCD are outlined.

**Trial Registration:**

Australian New Zealand Clinical Trials Registry ACTRN12611000321943; https://www.anzctr.org.au/Trial/Registration/TrialReview.aspx?id=336704 (Archived by WebCite at http://www.webcitation.org/70ovUiOmd)

## Introduction

Obsessive-compulsive disorder (OCD) is a common and highly debilitating condition that is considered to be among the most disabling of the psychological disorders [1]. OCD is characterized by intrusive and persistent thoughts, images, or urges (obsessions) that cause distress and lead to repetitive and often ritualistic behaviors (compulsions) intended to reduce threat or discomfort [2]. Cultural and geographically diverse clinical and epidemiological data have shown remarkable consistency with respect to both the presence and characteristics of OCD symptoms [3]. Without appropriate treatment, the course of the illness is understood to be chronic and lifelong [4].

Face-to-face individual and group-based cognitive behavioral therapy (CBT) has been shown to be a highly effective treatment for OCD [5,6], leading to significant improvements in functioning and quality of life [7,8]. Olatunji et al [9] assert that CBT is effective regardless of gender, baseline severity or symptom subtype, comorbidities, or treatment length. Greater pretreatment OCD severity has been associated with larger posttreatment effect sizes of face-to-face CBT, although not consistently across all studies [9-12]. While other psychological treatments have been applied to OCD, inclusive of progressive relaxation [13-16] and acceptance and commitment therapy [17], these interventions lack an extensive evidence base for their efficacy. Pharmacological treatments have also been found to be effective in treating OCD, particularly serotonergic agents, although cognitive behavioral treatments are more effective among outpatients with OCD [18].

Despite the existence of effective interventions, it is reported that there is around a 7-year delay from the individual’s first experiences of symptoms of OCD to their presenting for treatment [19,20]. People with the disorder may go undiagnosed for many years due to a failure of health professionals to recognize OCD [21] and because the individual does not disclose their experiences due to intense feelings of embarrassment and guilt [22-24]. For those who do present for help, access to treatment is poor. In particular, a shortage of appropriately qualified professionals (especially in geographically remote areas) along with long waitlists and individual financial constraints mean that only a small percentage of individuals with OCD receive CBT [24,25].

As a large proportion of people seek out mental health information from the internet [26,27], and in some cases feel more comfortable using technology than discussing their concerns in person [28-30], internet-based therapy could help bridge these gaps and make evidence-based treatments accessible and acceptable to individuals with OCD. Online treatments allow anonymity and are more accessible (particularly for geographically remote and rural areas), and depending on the model of therapy delivery, can be associated with reduced costs and allow the dissemination of standardized yet individualized treatments, providing the same content and skills as face-to-face equivalents [31].

While it is a relatively new area of investigation, initial findings suggest that iCBT is an effective modality for the treatment of OCD. A recent meta-analysis of remote CBT for OCD included 18 studies from which 7 were internet-administered interventions; however, only 2 studies were randomized controlled trials (RCTs) [32]. The author concluded that low- and high-intensity remote treatments for OCD lead to large magnitude improvements in OCD symptoms, are more effective than control conditions, and are not meaningfully different in efficacy from face-to-face treatment. In the largest RCT to date, Andersson et al [33] randomly allocated 101 participants to 10 weeks of therapist-guided iCBT or 10 weeks of online supportive therapy. The iCBT condition was associated with a significant reduction in OCD symptoms (Cohen *d*=1.55) as measured by the clinician-administered Yale-Brown Obsessive-Compulsive Scale (YBOCS) [34] compared to a medium within-group effect size (Cohen *d*=0.47) for the supportive therapy control. The results of iCBT were maintained at 4-month follow-up. Additionally, there was a large between-group effect size (Cohen *d*=1.12) on the YBOCS in favor of the iCBT condition. Finally, only 6% of participants in the control condition met criteria for clinically significant change compared with 60% of those receiving iCBT [33].

Similar results were demonstrated by Wootton et al [35], who found that 8-week courses of therapist-guided iCBT and therapist-guided bibliotherapy were both effective compared to a waitlist control condition (between-group effect sizes of Cohen *d*=1.57 and 1.40, respectively). Mahoney et al [36] conducted an RCT comparing clinically supervised technician-assisted iCBT to a treatment as usual control group. They found that iCBT was more efficacious than treatment as usual in reducing OCD severity, with iCBT demonstrating moderate-to-large effect sizes at posttreatment and 3-month follow-up depending on which severity measure was used and whether completers or the total sample were used in analyses. They reported that 54% of treatment completers no longer met diagnostic criteria for OCD at follow-up. A more recent study from Korea using an internet-based CBT program for OCD reported significant improvement in OCD severity (measured with the clinician-administered YBOCS) from pre- to posttreatment (Cohen *d*=1.64); however, the study did not include a comparison group and only analyzed completers (64% of the sample) [37].

While these iCBT studies reported effect sizes that are somewhat similar to face-to-face therapy [11], therapist contact times differ markedly. A standard course of face-to-face CBT would be around 15 to 30 one-hour sessions [38,39]. In contrast, Wootton et al [35] reported a mean total therapist time of 1.72 hours for therapist-guided bibliotherapy and 1.48 hours for iCBT. Andersson et al [33] reported a mean therapist contact time of 2.15 hours for iCBT, although this was significantly higher than for their control condition (0.28 hours). However, the between-groups difference remained significant after therapist contact was statistically controlled.

While these studies show promise for iCBT, there were limitations in terms of the study designs for the 2 RCTs conducted to date. First, in Andersson et al [33], the control group comprised nondirective supportive therapy and lacked online modules. Similarly, the control condition in the study by Wootton et al [35] included no modules and no therapist time allocations, although a therapist-directed bibliotherapy group was included as an active control condition. As such, neither of the control groups in the 2 RCTs matched the active online treatment components in terms of format (ie, online self-help information), therapist contact, medium (ie, audiovisual content, downloadable worksheets, and other content), or therapeutic processes (eg, homework). Hence, research still needs to establish how iCBT compares to comparator conditions that serve as bona fide controls relative to active treatment.

Building upon these studies, our study aimed to evaluate therapist-assisted iCBT for OCD as compared to an analogous active control (therapist-assisted internet-based progressive relaxation therapy [iPRT]), allowing us to identify the additive effects of iCBT beyond the nonspecific consequences of anxiety management or expectation of change. At the completion of the iPRT, that group was administered the iCBT program. This paper reports pre-post iCBT treatment outcomes. Specifically, we used a new online CBT program for OCD and compared it to iPRT based on the protocol developed by Bernstein et al [40]. As justification, the most recent evaluation using manualized PRT has shown it to be effective and credible in treating OCD [17], although its efficacy has not been a consistent finding in the past [41,42]. More recently, an online applied relaxation program based on Öst [43] and very similar in content and structure to that of Bernstein et al [40] was found to be effective in the management of anxiety in panic disorder [44].

Consistent with our published study protocol [45], this paper reports primary outcomes of the study, namely pre-post change in OCD severity ratings and the proportion of participants experiencing clinically significant change. A second paper will report secondary outcomes. It was hypothesized that both groups would experience significant improvements in symptoms of OCD from pre- to posttreatment using an iPRT, with significantly greater improvements in iCBT. Specifically, we hypothesized that both the iCBT and iPRT groups would experience a reduction in symptom severity from pre- to postintervention with significantly greater improvement for the iCBT group. Participants who completed the control condition were anticipated to experience a further significant drop in OCD symptoms after completing iCBT. It was further expected that the proportion of participants experiencing clinically significant change in symptom severity would be greater for iCBT than iPRT.

Finally, the influence of sociodemographic and clinical variables was used to predict improvements in YBOCS scores. Given mixed results from previous studies and meta-analyses of face-to-face and remote CBT for OCD, we do not offer directional hypotheses regarding predictors of symptom improvement. Furthermore, evidence for predictors and moderators of outcome in internet-based interventions is still very limited [46]. Hence, we merely explored whether treatment gains were predicted by clinical variables (eg, symptom severity, depression scores, medication status) and sociodemographic measures (eg, gender, marital status).

## Methods

### Design

The study protocol is described elsewhere, inclusive of details about power analyses and measures [45]. There were minimal deviations from the protocol in the conduct of the trial; the major exception was that, due to difficulties in contacting and engaging participants, we were not able to undertake all the intended posttreatment assessments (eg, posttreatment structured diagnostic interviews). Rather, we focused on conducting as many telephone-administered OCD severity (YBOCS) interviews as possible. Subsequently, analyses are based on OCD severity and associated recovery (as defined below). Our evaluation framework was based on the work of Öst [47], who has developed a psychotherapy outcome study methodology rating form. In summary, the study conformed to Consolidated Standards of Reporting Trials (CONSORT) requirements [48]; participants were randomized post baseline interview into the active treatment (iCBT) or control condition (iPRT) using an independent automated computer-generated randomization sequence that could not be forecast or modified by the researchers. Stratified randomization was used to achieve gender balance between groups. The randomization was coordinated by an independent statistician. This paper compares pre- to posttreatment OCD outcomes using between- and within-group results. Posttreatment assessments were blind to treatment condition.

### Participants, Recruitment, and Measures

**Figure 1:**
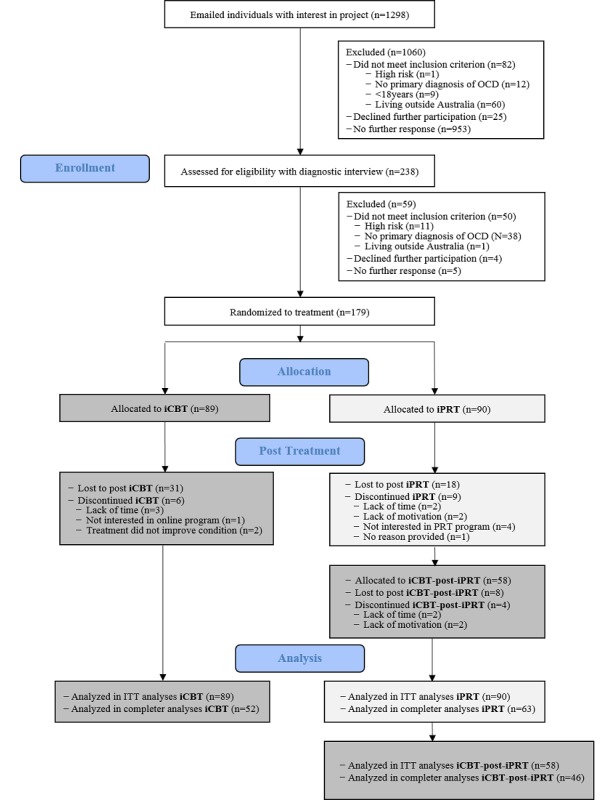
Consolidated Standards of Reporting Trials flow diagram of study. iCBT: internet-based cognitive behavioral therapy; iPRT: internet-based progressive relaxation training; OCD: obsessive-compulsive disorder.

Participants were recruited by referral from primary care physicians and mental health professionals and through self-referral. Information about the study was publicized on a webpage, on an affiliated online mental health treatment webpage [49], on YouTube and via online advertisements on Facebook, and in mail-outs to Australian mental health professionals. Recruitment for the trial started in early 2013, preintervention assessments commenced in July 2013, and the last postintervention assessment was conducted in July 2014. From 1298 people who registered initial interest either online or via telephone, 238 participants provided consent and completed the pretreatment interview via telephone. Inclusion criteria were: (1) Australian resident; (2) aged 18 years or over; (3) fit the then-current *Diagnostic and Statistical Manual of Mental Disorders, 4th Edition, Text Revision* (DSM-IV-TR) criteria for a primary diagnosis of OCD [50] where hoarding was not the primary symptom as assessed by the Structured Clinical Interview for DSM-IV Axis 1 Disorders, Clinician Version (SCID-CV) [51]; (4) no current psychosis, substance abuse, head injury, or neurological disorder; (5) no current active suicidal ideation or, if high risk (eg, history of suicidal behavior) then had appropriate psychosocial supports during the course of the trial; and (6) access to a computer. Participant flow is shown in Figure 1. Participant numbers were consistent with those anticipated from our initial power analysis [42] that expected a large effect for iCBT, a moderate effect size for iPRT, power at 80%, and a standard deviation of 5 for the distribution of mean change with significance set at 5% (alpha=.05). We did not collect the planned additional 20% of participants as the research funding period was ending. We ceased recruitment of participants after reaching numbers that were anticipated from the power analyses.

This paper focuses on the major outcome measures. Participants were enrolled by the study coordinator who allocated participants to a pretreatment assessor, the treatment condition on the basis of the randomization process, and an e-therapist. Applicants who met inclusion criteria (N=179) were assessed for baseline OCD severity with the telephone-administered clinician-rated YBOCS [34] and comorbid diagnoses were assessed with the SCID-CV [51]. The SCID-CV is the gold standard in structured diagnostic interviews, and the clinician-rated YBOCS has shown excellent reliability (eg, interrater reliability) and convergent validity with other measures of OCD [52]. Internal consistency for the YBOCS in our sample was acceptable (Cronbach alpha=.75). In addition to assess baseline depressive and anxiety symptoms and consistent with our previous research [53], the Hamilton Depression Rating Scale (HAM-D) [54] and the Hamilton Anxiety Rating Scale (HAM-A) [55] were used. Internal consistencies of the HAM-D (Cronbach alpha=.78) and HAM-A (Cronbach alpha=.79) were acceptable. Two questions were used to assess baseline treatment expectancies (“I believe this treatment is likely to be effective” and “I believe this treatment is likely to result in permanent improvement”); the questions were scored on a 5-point Likert scale ranging from strongly disagree (0) to strongly agree (4), and a composite score of the 2 items was calculated (Spearman-Brown coefficient=.80) to assess treatment expectancies in a subsample across the 2 intervention groups (96/179, 53.6% of the total sample).

Assessors (n=26) were either licensed psychologists or supervised students undertaking a masters or professional doctorate in clinical psychology. An experienced clinical psychologist trained all assessors in psychiatric diagnosis and structured interviews and reviewed all case inclusions. All assessors were trained through the use of videos and clients from the University Psychology Clinic until there was 100% consistent agreement with the senior assessor. Assessors were blind to treatment condition allocation. To examine the reliability between assessors, intraclass correlations (ICC) of YBOCS total scores were calculated; there was a high agreement between assessors (ICC=.94, 95% CI 0.68-0.99).

The overall sample was 65.7% female (117/179) and on average aged 33.4 (SD 9.9) years. The majority of participants had at least a bachelor degree (98/179, 54.7%) and were in de facto relationships or were married (87/179, 48.6%). On average, the sample reported having experienced OCD symptoms for almost 14 years (mean 13.7 [SD 0.8] years, range 1-49), with a current YBOCS rating of 21.94 (SD 0.49) and range 7-36. Almost half of the sample (84/179, 46.9%) reported moderate OCD symptoms (YBOCS 16-23), while around another third (62/179, 34.6%) indicated severe symptoms (YBOCS 24-31); the other participants reported either mild (YBOCS 8-15; 21/179, 11.7%) or extreme OCD symptoms (YBOCS 32-40; 9/179, 5.0%). At the start of the trial, approximately one-third (66/179, 36.9%) of participants were receiving another form of psychological treatment and two-thirds (124/179, 69.3%) were on medication, with no changes in these allowed during the trial and randomization meaning there were no differences at baseline between the 2 conditions (see Tables 1 and 2).

### Interventions

Both intervention groups used their personal computer or laptop to access the intervention modules; weekly emails by the e-therapist were sent to the email address provided in the baseline assessment. There was no charge for treatment. Participants were not offered any direct incentives but had the opportunity to enter a raffle to win 1 of 3 tablet computers if they completed all assessments. This incentive was only offered midway through the trial as a way to increase questionnaire completion. Note that the incentive was not provided to complete the program but rather to complete the assessments. Previous research and our experience with delivery of treatment programs has indicated that assessment completion does not reflect program completion, with a large portion of individuals completing programs but not the final assessment [56,57].

**Table 1:** Sociodemographic characteristics of the internet-based cognitive behavioral therapy (iCBT) and internet-based progressive relaxation therapy (iPRT) groups.

Characteristics		iCBT group			iPRT group			Test		*P* value
		n^a^	Value	n^a^		Value				
Age, years, mean (SD)		88	32.59 (9.86)	87		34.23 (9.88)	*F*_1,173_=1.21		.27	
# of children, mean (SD)		81	0.79 (1.17)	84		0.95 (1.33)	*F*_1,163_=1.09		.41	
**Gender, n (%)**		89		89			χ^2^_1_=0.03		.88	
	Male		31 (34.8)			30 (33.7)				
	Female		58 (65.2)			59 (66.3)				
**Education, n (%)**		84		87			χ^2^_2_=2.02		.37	
	At most secondary school		38 (45.2)			35 (40.2)				
	Bachelor degree		33 (39.3)			31 (35.6)				
	Postgrad degree		13 (15.5)			21 (24.1)				
**Marital status, n (%)**		82		82			χ^2^_2_=1.73		.42	
	Married/de facto		43 (52.4)			44 (53.7)				
	Never married		35 (42.7)			30 (36.6)				
	Other		4 (4.9)			8 (9.8)				
**Working, n (%)**		67		68			χ^2^_2_=0.61		.74	
	No		11 (16.4)			13 (19.1)				
	Part-time		18 (26.9)			21 (30.9)				
	Full-time		38 (56.7)			34 (50.0)				

^a^Sample size varies due to missing data; N=179 randomized for treatment, n=89 allocated to iCBT, n=90 allocated to iPRT.

Both conditions comprised 12 modules delivered online over a 12-week period. Participants were encouraged to complete 1 module per week for the duration of treatment but all aspects of the program could be accessed from the beginning. Both conditions included online psychoeducational information, weekly homework tasks, downloadable worksheets, and audio files. All participants received a single email per week from a remote therapist irrespective of how many emails participants had sent; participants were free to email as often as they wished. If no emails were received from participants, 2 further reminder emails were sent. Emails to participants aimed to monitor progress, provide support and encouragement, and assist in tailoring the treatment to participants’ problems, in line with the online content of their allocated condition. Therapist time spent on emails ranged from 0 to 15 minutes per week per participant during the treatment phase for each of the iCBT and iPRT conditions. If participants did not log into the treatment modules the previous week, they were reminded to do so by their e-therapist in the weekly emails.

Therapists (n=10) were either psychologists or students undertaking a masters or professional doctorate in clinical psychology and underwent an online training module for e-therapists working within the Mental Health Online platform [49]. An experienced clinical psychologist provided biweekly supervision of therapists to maintain adherence to intervention-relevant treatment integrity and ensure clinicians preserved adherence to the time limits expected for responding to emails.

iCBT (see Table 3 for program content) comprised 12 modules with psychoeducation about OCD, anxiety, and an introduction to CBT, along with mood and behavior management strategies. Exposure and response prevention (ERP) strategies were covered along with instructions and examples on how to construct a range of hierarchies and how to conduct ERP. Cognitive therapy techniques (eg, cognitive restructuring, behavioral experiments) targeting OCD-specific cognitive styles (such as inflated responsibility or overestimation of threat, importance/control of thoughts, perfectionism, and uncertainty intolerance beliefs) were also provided. Relapse preventions strategies (eg, problem solving, risk identification) were introduced toward the end of the treatment. The importance of daily practice was emphasized with a focus on enablers of and barriers to maintenance of CBT. Audiovisual content, case stories, demonstrations of techniques, downloadable audio content and worksheets, and expert commentary were provided throughout the program.

The control condition (see Table 3 for program content) was a 12-module iPRT program adapted from Bernstein et al [40]. Participants received basic information about OCD, on the relationship of OCD to anxiety, and about the use of relaxation as a way to manage anxiety. Individuals were taught to relax specific muscle groups while paying attention to sensations associated with both being tense or relaxed. Individuals were instructed on how to achieve a state of deep relaxation in increasingly shorter periods and control excess tension in stress-inducing situations. Participants sequentially tensed and released muscle groups in order to achieve maximum states of relaxation, with the number of muscle groups decreasing over the 12 modules. Participants had access to downloadable audio and written material to guide their progressive relaxation training. ERP, cognitive therapy, and other CBT elements were not included in the iPRT program. Participants randomized into iPRT were aware that they would have the option of taking up iCBT at the end of their allotted condition (ie, iCBT post-iPRT).

### Statistical Analyses

All analyses were conducted with SPSS Statistics version 22 (IBM Corp) using the YBOCS as the primary outcome measure. Group differences in demographic data and pretreatment measures were analyzed with 1-way analyses of variance and chi-square tests. Mixed-models analyses employing an autoregressive covariance structure and restricted maximum likelihood estimation were used to analyze changes in YBOCS scores from pre- to posttreatment in an intention-to-treat (ITT) analysis while controlling for age due to a significant age effect for attrition. Effect sizes (Cohen *d*) with 95% standardized confidence intervals were calculated for both within-group and between-group effects based on observed means and the pooled standard deviations.

The following criteria of clinical significance were used: a person was deemed to have made a reliable improvement if, at pretreatment, a score of 16 or greater was reported on the YBOCS and more than 6 units improvement were observed (ie, approximately 1 standard deviation) during treatment. A YBOCS score of 16 has traditionally been regarded as the cutoff score to indicate clinical significance in OCD trials [23], although some literature regards a cutoff of 14 as more appropriate [58,59]. We took the traditional approach (ie, using a cutoff of 16 on the YBOCS) in our main analysis but also examined the data using the more conservative approach.

Linear regression analyses were used to examine the effect of pretreatment YBOCS levels on the improvement of OCD severity for the 2 treatment groups separately. Additional regression analyses were performed using the total sample to test effects of sociodemographic variables and indicators of disability on YBOCS improvement levels.

**Table 2:** Baseline clinical characteristics of the internet-based cognitive behavioral therapy (iCBT) and internet-based progressive relaxation therapy (iPRT) groups.

Characteristic		iCBT group		iPRT group		Test	*P* value
		n^a^	Value	n^a^	Value		
YBOCS^b^, mean (SD)		89	22.58 (5.53)	88	22.22 (5.76)	*F*_1,175_=0.18	.67
Years since onset of OCD^c^, mean (SD)		67	12.28 (9.16)	71	15.01 (10.49)	*F*_1,136_=2.64	.11
GAF^d^ scale, mean (SD)		80	57.41 (8.00)	74	59.34 (10.74)	*F*_1,152_=1.63	.20
HAM-D^e^ scale, mean (SD)		88	10.78 (6.25)	87	9.98 (5.70)	*F*_1,173_=0.79	.38
HAM-A^f^ scale, mean (SD)		88	15.19 (8.82)	86	14.09 (7.61)	*F*_1,172_=0.78	.38
# hospitalizations, mean (SD)		85	0.27 (0.79)	86	0.14 (0.62)	*F*_1,170_=2.44	.12
Treatment expectancy, mean (SD)		50	2.45 (0.71)	46	2.38 (0.76)	*F*_1,94_=0.21	.65
**Other current psychological treatment, n (%)**		76		75		χ^2^_1_=0.83	.36
	No		40 (52.6)		45 (60)		
	Yes		36 (47.4)		30 (40)		
**Current medication, n (%)**		85		85		χ^2^_1_=0.48	.49
	No		25 (29.4)		21 (24.7)		
	Yes		60 (70.6)		64 (75.3)		
**Comorbidity, n (%)**		82		78		χ^2^_1_=0.13	.72
	No		19 (23.2)		20 (25.6)		
	Yes		63 (76.8)		58 (74.4)		

^a^Sample size varies due to missing data; N=179 randomized for treatment, n=89 allocated to iCBT, n=90 allocated to iPRT.

^b^YBOCS: Yale-Brown Obsessive-Compulsive Scale.

^c^OCD: obsessive-compulsive disorder.

^d^GAF: Global Assessment of Functioning.

^e^HAM-D: Hamilton Depression Rating Scale.

^f^HAM-A: Hamilton Anxiety Rating Scale.

**Table 3:** Content of the intervention modules for therapist-assisted internet-based cognitive behavioral therapy and progressive relaxation therapy.

Intervention and modules		Contents
**iCBT^a^**		
	1-3	Psychoeducation about OCD^b^ and anxiety Introduction to CBT Mood management strategies (eg, activity scheduling)
	4-6	Exposure and response prevention strategies (eg, construction of fear hierarchies)
	7-9	Cognitive therapy techniques (eg, cognitive restructuring) targeting OCD-specific cognitive styles (eg, inflated responsibility or overestimation of threat, importance of control of thoughts)
	10-12	Relapse preventions strategies (eg, problem solving, risk identification, contingency management, and mindfulness techniques)
**iPRT^c^**		
	1-3	Psychoeducation about OCD and anxiety Introduction to PRT Sequential tensing and releasing of 16 muscle groups
	4-5	Sequential tensing and releasing of 7 muscle groups
	6-7	Sequential tensing and releasing of 4 muscle groups
	8-11	Releasing of muscle groups without tension component (relaxation through recall)
	12	Mental summary of previously learned techniques

^a^iCBT: internet-based cognitive behavioral therapy.

^b^OCD: obsessive-compulsive disorder.

^c^iPRT: internet-based progressive relaxation therapy.

## Results

### Examination of Covariates

All analyses were conducted with and without using pretreatment YBOCS, depression, and anxiety scores as covariates. Inclusion of covariates did not change the pattern of results; thus, results not including covariates are reported. In addition, Pearson correlations were conducted between pretreatment anxiety and depression and posttreatment YBOCS scores, revealing only a marginal influence of the covariates on posttreatment OCD severity; HAM-A *r*_119_=.19 and HAM-D *r*_120_=.14. Results were compared with those obtained using a completer sample. For the completer analyses, cases were used only if pre- and postintervention OCD severity data (ie, YBOCS) was available. Little’s MCAR test supports the assumption of data missing completely at random for pre- and postintervention YBOCS, HAM-D, and HAM-A across the 2 conditions indicated that data were missing at random (*χ*^2^_1_=1.02, *P*=.31).

### Baseline Differences and Completers

There were no significant differences between the groups on demographic or mental health variables (all *P*>.05; Tables 1 and 2). In addition, there were no significant differences in treatment expectancies (*F*_1,94_=0.21, *P*=.64; see Tables 1 and 2).

From the iCBT group, 7% of participants (6/89) discontinued the treatment compared to 10% of participants (9/90) in the iPRT group; this difference was not statistically significant (*χ*^2^_1_=0.62, *P*=.43). Common reasons offered for ceasing the treatment in both conditions were other life commitments: 2 participants from the iCBT group dropped out because they did not find the treatment helpful, and 4 participants from the iPRT group indicated that the treatment was not specific to OCD, not effective, or that they would prefer to receive the CBT intervention (see Figure 1). Combining participants who discontinued treatment and/or did not complete the posttreatment assessment, there were more such participants in the iCBT group (37/89, 42%) compared to the iPRT group (27/90, 30%); however, this difference was not statistically significant (*χ*^2^_1_=2.61, *P*=.11). No significant differences (*P*>.05) were found between participants who did and did not complete posttreatment assessments on gender, education, marital status, number of hospitalizations, pretreatment YBOCS scores, whether participants had received any kind of treatment or medication in the month prior to commencing the study, or baseline treatment expectancies. However, there was a significant difference on age, with younger participants less likely to complete the posttreatment assessment (*F*_1,173_=4.14, *P*=.04), making an ITT analysis advisable. Posttreatment completion rates increased on average by 3.4% for each year of age (95% CI 0.1%-6.7%).

### Pre-Post Treatment Improvements

**Figure 2:**
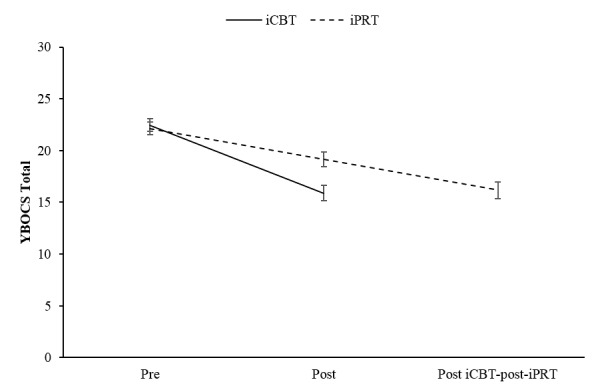
Pre- and post-internet–based cognitive behavioral and progressive relaxation therapy estimated means including standard error bars. iCBT: internet-based cognitive behavioral therapy, iPRT: internet-based progressive relaxation therapy, YBOCS: Yale-Brown Obsessive-Compulsive Scale.

Means and standard deviations at pre- and posttreatment for the YBOCS are shown in Table 4, while Figure 2 shows the pattern of results. In order to test whether participants in the iCBT group showed greater improvement in OCD severity compared to improvements in the iPRT group, changes in YBOCS scores from pre- to posttreatment across the 2 treatment groups (ie, iCBT vs iPRT) were analyzed while controlling for age.

No negative experiences were reported at posttreatment by participants for the iCBT or iPRT interventions. Results for the ITT sample showed a significant time × group interaction effect (*F*_1,114_=11.75, *P*=.001) and a significant main effect for time (*F*_1,148_=83.52, *P*<.001); however, there was no significant main effect of group (*F*_1,180_=3.58, *P*=.06), suggesting that both treatment groups improved over time but the iCBT group showed greater improvement compared to the iPRT group. These results were replicated in the completer sample: time × group interaction effect (*F*_1,107_=6.91, *P*=.01), main effect for time (*F*_1,107_=110.05, *P*<.001), and main effect for group (*F*_1,109_=3.61, *P*=.06).

Paired *t* tests were conducted to test within-group improvements on the YBOCS. A *t* test comparing pre- and post-iCBT scores was statistically significant (*t*_56_=7.90, *P*<.001); on average participants in the intervention group (iCBT) improved by 6.40 units on the YBOCS (95% CI 4.78-8.03). A second *t* test comparing pre- and post-iPRT scores was also significant (*t*_66_= 3.92, *P*<.001); on average participants in the control group (iPRT) improved by 2.90 units on the YBOCS (95% CI 1.43-4.38). There were large improvements in OCD severity from pre- to posttreatment in the iCBT group and medium-to-large improvements in the iPRT group (see Table 4).

### Post-Internet–Based Progressive Relaxation Therapy Improvement

In order to compare overall improvement in OCD severity between the iCBT cohort and the group undertaking iPRT followed by iCBT (iCBT–post-iPRT), changes in YBOCS scores from pretreatment (either condition) to post-iCBT treatment were analyzed while controlling for age: ITT analysis indicated a significant main effect for time (*F*_1,152_=116.31, *P*<.001); however, there was no significant interaction effect for time × group (*F*_1,152_=0.23, *P*=.63), suggesting that the combined iPRT/iCBT condition was no more effective than the iCBT condition on its own.

A *t* test comparing pre-iCBT and post-iCBT scores in the group that had previously received the iPRT intervention (iCBT–post-iPRT) was significant (*t*_48_=4.03, *P*<.001); after having received the iPRT treatment, participants who continued with the iCBT treatment improved on average by 3.14 units on the YBOCS (95% CI 1.56-4.72). Moderate improvements were found from pre-iCBT to post-iCBT for the group that had previously received the iPRT treatment (iCBT–post-iPRT) (Table 5). Figure 2 presents the estimated means across the 2 treatments including standard error bars.

### Reliable Improvement and Reliable Recovery

Using the previously discussed definition for reliable improvement (ie, at least a 6-unit YBOCS change) and reliable recovery (reliable improvement plus YBOCS below 16 at posttreatment), for the ITT analysis there were statistically significant differences between the 2 treatments for both of these variables (Table 6). Of the people with YBOCS scores of at least 16 prior to the iCBT treatment, 49% (25/51) showed an improvement of at least 6 units and 33% (17/51) made a reliable recovery. For the iPRT treatment, 29% (16/55) of the people with a YBOCS score of at least 16 prior to treatment showed an improvement of more than 6 units, however, only 11% (6/55) made a reliable recovery. Note that only 1 participant with a YBOCS score initially below 16 showed an improvement of at least 6 units. Using more conservative criteria defined by Fisher and Wells [58] (YBOCS cutoff <14 and reliable change of YBOCS >10), 18% (9/51) of the iCBT were considered to have been reliably recovered compared to 6% (3/55) of the iPRT group; this difference was statistically significant (*χ*^2^_1_=3.92, *P*=.048).

We also identified 4 participants who had deteriorated at posttreatment (ie, deterioration of at least 6 units in YBOCS severity scores and an overall posttreatment YBOCS score above 16): 3 participants in the iPRT and 1 participant in the iCBT condition. Demographic variables were comparative to the rest of the sample (eg, age, education, gender); however, pretreatment severity scores tended to be lower compared to the rest of the sample (YBOCS mean 18.75 [SD 6.50], HAM-D mean 8.50 [SD 7.14], HAM-A mean 11.00 [SD 10.03]).

### Prediction of Obsessive-Compulsive Disorder Severity Improvement Using Pretreatment Characteristics

Using regression analyses, the effect of pretreatment YBOCS scores on levels of improvement was compared for the iCBT and iPRT treatments. For the iCBT treatment, there was a significant positive relationship between pretreatment severity and improvement levels (*t*_55_=2.37, *P*=.02) with an average improvement of 0.58 units for each additional unit on the pretreatment YBOCS. For the iPRT treatment, there was also a significant positive relationship between pretreatment severity and improvement levels (*t*_65_=2.59, *P*=.01) with an average improvement of 0.43 units for each additional unit for the pretreatment YBOCS. However, this difference in average improvements between the 2 treatments was not significant (*t*_121_=0.90, *P*=.37). These results suggest that both treatments are more effective for individuals with higher initial OCD severity.

**Table 4:** Changes in the Yale-Brown Obsessive-Compulsive Scale scores from pre- to postintervention.

Analysis			Preintervention					Postintervention					Effect size, Cohen *d* (95% CI)		
			mean (SD)^a^		SE^b^		mean (SD)			SE		Within			Between
**ITT^c^ analysis**															0.55 (0.18-0.91)
	iCBT^d^ YBOCS^e^ score (n=89)	22.44 (5.36)		.61		15.86 (5.65)			.74		1.05 (0.72-1.37)				
	iPRT^f^ YBOCS score (n=90)	22.13 (5.73)		.62		19.15 (6.45)			.69		0.48 (0.22-0.73)				
**Completer analysis**															0.57 (0.18-0.95)
	iCBT YBOCS score (n=52)	22.18 (5.61)		.81		15.26 (5.01)			.81		1.24 (0.87-1.60)				
	iPRT YBOCS score (n=63)	22.64 (6.02)		.62		18.49 (6.35)			.76		0.78 (0.49-1.08)				

^a^Estimated marginal mean and standard deviation based on a mixed model analysis.

^b^SE: standard error.

^c^ITT: intention-to-treat.

^d^iCBT: internet-based cognitive behavioral therapy.

^e^YBOCS: Yale-Brown Obsessive-Compulsive Scale.

^f^iPRT: internet-based progressive relaxation therapy.

**Table 5:** Changes in the Yale-Brown Obsessive-Compulsive Scale scores for participants in the progressive relaxation versus cognitive behavioral therapy groups.

Analysis	Post-iPRT^a^		Post-iCBT (post-iPRT)^b^		Effect size (Within), Cohen *d* (95% CI)	
	mean (SD)^c^	SE^d^	mean (SD)	SE		
ITT^e^ analysis (n=58)	19.15 (6.45)	.69	16.16 (6.89)	.81	0.55 (0.21-0.88)	
Completer analysis (n=46)	18.49 (6.35)	.76	15.26 (5.01)	.78	0.55 (0.22-0.88)	

^a^Post-iPRT: postintervention internet-based progressive relaxation therapy.

^b^Post-iCBT (post-iPRT): postintervention internet-based cognitive behavioral therapy (post progressive relaxation therapy).

^c^Estimated marginal mean and standard deviation based on a mixed model analysis.

^d^SE: standard error.

^e^ITT: intention-to-treat.

**Table 6:** Reliability of improvement and recovery for intention-to-treat analysis at posttreatment.

Treatment	Reliable improvement			Reliable recovery		
	n (%)	χ^2^	*P* value	n (%)	χ^2^	*P* value
iCBT^a^ (n=51)	25 (49)	4.43	.04	17 (33)	7.83	.01
iPRT^b^ (n=55)	16 (29)	N/A^c^	N/A	6 (11)	N/A	N/A

^a^iCBT: internet-based cognitive behavioral therapy.

^b^iPRT: internet-based progressive relaxation therapy.

^b^N/A: not applicable.

Finally, separate regression analyses were performed using the total sample and the iCBT and iPRT groups. In the first regression model using the total sample, sociodemographic variables (gender, age, number of children, education, and marital status) were used to predict improvements in YBOCS scores. None of the sociodemographic variables explained a significant amount of variance in YBOCS improvement scores (*F*_5,96_=0.42, *P*=.84). In a second regression model, indicators of disability (pretreatment global assessment of functioning, depression, anxiety scores, medication, number of hospitalizations) were used as predictors for YBOCS change scores in a subgroup of participants for whom full disability data sets were available, and none of these indicators explained a significant amount of variance in YBOCS improvement scores (*F*_5,95_=2.06, *P*=.08). However, note that due to an administrative error, sociodemographic and disability data were not available for all participants. Nonetheless, there were no differences in pretreatment YBOCS scores between participants with and without missing sociodemographic (*t*_175_=1.28, *P*=.20) and disability data (*t*_175_=0.62, *P*=.53) used in the regression analyses.

In line with the results for the total sample, none of the sociodemographic variables or the indicators of illness severity were significant predictors for YBOCS change scores in the iCBT or iPRT group except pretreatment depression scores predicting YBOCS improvement scores in the iPRT group (*t*_45_=2.41, *P*=.02), suggesting that participants with higher initial depressive symptoms showed greater YBOCS improvement scores; with every point on the HAM-D, participants in the iPRT group improved on average by 0.5 points more on the YBOCS.

## Discussion

### Principal Findings

Access to evidence-based treatment for psychological disorders can be facilitated by the digital revolution and the advent of online CBT, which is seen as an important component of contemporary mental health policies [60]. Such access is particularly important for a mental health condition such as OCD, which requires specialized treatment and is prone to effects of low help-seeking due to shame and stigma associated with the disorder [61]. This RCT aimed to evaluate the effect of therapist-assisted iCBT for OCD compared to an analogous active iPRT condition. This paper reports pre-post findings, with upcoming papers reporting follow-up findings and patterns of use associated with outcome.

Our findings demonstrate the large magnitude effect of a therapist-assisted iCBT for OCD. The study also established that a structured iPRT was efficacious, albeit less so than the iCBT intervention. While the addition of iCBT sequentially immediately following iPRT led to further significant symptom amelioration, the combined treatment was no more efficacious than iCBT alone. A similar pattern of results was found when we examined reliable improvement (ie, at least a 6-unit YBOCS change) and reliable recovery (reliable improvement plus YBOCS below 16). The iCBT condition was superior to iPRT, with around half of those in the iCBT treatment making a reliable improvement and a third making a reliable recovery compared to only 29% and 11%, respectively, in the iPRT condition. An exploration of predictors of treatment response found that pretreatment OCD symptom severity was the only significant predictor of change. No sociodemographic or psychopathology severity variables predicted improvement. The small number of participants who deteriorated presented with an interesting profile; they tended to have lower severity scores on OCD, depression, and anxiety. This is contrary to what one might expect; however, previous literature has not generally reported deterioration statistics, although the relevant samples have been very small. The characterization of participants undertaking internet-based therapies who deteriorate is an important future research question and will require greater power, given the small numbers.

The magnitude of symptom amelioration is largely commensurate with previous studies of iCBT treatment indicating large effect sizes [32,62], although posttreatment YBOCS scores in this study were slightly higher than those reported in previous trials, while pretreatment YBOCS were either on par or slightly higher [33,35]. Our study used a more rigorous design by including an active control group that was comparable in terms of amount of content, prescribed therapist time, and mode of delivery, providing strong support for the efficacy of the specific CBT interventions over and above the effect of more general factors such as therapist support and time in treatment. While an inactive control might have been a useful addition from a design perspective, ethical considerations precluded this. Note that previous comparisons of iCBT against treatment as usual indicated no significant effects for the inactive control [36]. Overall, the findings support the notion that iCBT is an effective treatment for OCD, a disorder characterized particularly by shame, stigma, delayed help-seeking, and poor access to expert treatment [21-25].

Recovery figures in this study of around a third for iCBT were in the lower range compared to those reported in previous face-to-face and online treatment studies [33,35,58,63,64], although the expected superiority of iCBT over iPRT in recovery was supported. The slightly higher posttreatment YBOCS scores in our study certainly account for our findings. The nature of participants and recruitment strategies may partially explain these findings. Participants in this study were chronic in their presentation as indicated by over three-quarters reporting comorbidity, around 70% already on medications, and around half engaged in other forms of psychotherapy. While, on average, onset of OCD was reported as around 12 to 15 years, this compares to around 18 years in 1 similar study with better recovery rates [33]. Nonetheless, the literature generally asserts that around 25% to 70% of participants experience clinically significant change [33,35,58,63,64], although such definitions of recovery vary greatly between studies in the OCD area, and there has been a call to develop standard criteria [6,11]. One way around this is to use structured diagnostic interviews to assess recovery from diagnostic status, as originally intended, but participants were reticent to comply with the time required to undertake long interviews at posttreatment. Given revised DSM-5 criteria for OCD, future research will need to incorporate updated diagnostic interviews to assess recovery status.

Our results were based on ITT analyses of YBOCS severity data with available data biased toward older participants. It is possible that younger people are more transient or less likely to make themselves available for posttreatment assessments or they were impacted differentially more by the burden of multiple detailed assessments. Alternatively, they may have recovered more, may have deteriorated and dropped out of the study, or may have experienced decreased motivation to participate in further assessments due to the generally longer treatment (12 modules over 12 weeks for our study compared to 8 and 10 weeks for 5 and 10 modules in previous RCTs [35,33]). Nonetheless, treatment completion was high overall, supporting the degree to which internet-based treatments engage participants. Hence, while it is possible that effects were over- or underestimated, the large magnitude effect of iCBT is consistent with prior findings.

Previous literature has found mixed results with respect to predictors of treatment response [6,9-11,65]. On the one hand, that sociodemographic variables and disability did not predict outcome in this trial of an online treatment for OCD was encouraging in terms of suggesting the general utility of the intervention. However, it also leaves us none the wiser as to predicting which demographic group might most benefit from this intervention. Future research will need to examine participants from a broader range of social, educational, and cultural backgrounds and disability, chronicity, and comorbidity profiles in order to assess whether there are variables tied to differential effectiveness of the intervention. Future studies may also need to examine predictions away from the more controlled context of RCTs.

That greater pretreatment OCD symptom severity is a significant predictor of better outcome in internet-based and face-to-face treatments is consistent with much of the previous literature [10-12,65]. This finding is no surprise as those with greater symptom severity have greater scope for larger magnitude symptom amelioration. Nonetheless, it was encouraging to note that the results suggest that both iCBT and iPRT were effective for those with higher initial OCD severity. On the surface, this may contrast with a generally held expectation, embedded within national mental health policies and practice guidelines [60], that online treatments should be used to target only mild severity presentations. However, an examination of mean severity scores across studies suggests that those presenting for online treatments are generally in the mild-to-moderate severity range, with mean clinician-rated YBOCS scores of around 21 to 25 [32], in contrast to a slightly higher but broader range of scores (17 to 29) for face-to-face individual and group treatment studies [11].

Future research will benefit from further examining the differential effectiveness of treatment for different OCD symptom profiles. Little previous research has examined differences in outcome among different OCD subtypes, although the emergence of new measures such as the Dimensional Obsessive-Compulsive Scale [66] will allow such examination. Examination of subtype performance in online studies would be particularly useful. For instance, do individuals with obsessional presentations respond less well than do those with compulsions? Are individuals with contamination and washing presentations more responsive to the structured approach that is inherent in online treatments than are, say, individuals with obsessional checking or those with obsessional slowness? Such insights would allow the development of more targeted treatment guidelines.

One of the more interesting findings from this trial was that iPRT is moderately efficacious if embedded within a framework of managing anxiety in OCD situations, although results may have been influenced by a biased sample that maintained adherence in order to undertake iCBT. While older trials had previously concluded that the efficacy of PRT was limited for OCD [41], a more recent study by Twohig et al [17] reported that PRT had a large magnitude effect in an RCT, raising the possibility that PRT is more efficacious than had been believed in the treatment of OCD. For ethical and practical purposes, we embedded the PRT within the context of coping with anxiety in OCD-relevant situations rather than merely engaging participants in decontextualized progressive muscular relaxation training, meaning that participants may have found the information to be more personally relevant than in some PRT protocols. A recent online applied relaxation program was found to be effective in the management of anxiety in panic disorder [44]; hence, the anxiety management component of iPRT may have moderate but significant specific benefits in OCD.

Regardless of the reasons, while there was satisfactory adherence and relatively good effects for iPRT, these were still limited relative to iCBT, and the combined iPRT/iCBT did not perform any better than iCBT alone. Nonetheless, while guidelines to use CBT over interventions such as PRT are supported by the current findings, iPRT may prove useful in cases where the individual’s capacity or willingness to undertake CBT is compromised. Future studies could examine the characteristics of affected individuals who respond specifically to the use of iPRT.

Future research will also need to pay greater attention to mechanisms of change. What is the impact on outcome of specific components of treatment protocols, treatment length or integrity, amount of time spent by assessors in interviewing participants, content of messages sent by therapists in responding to participant emails, and markers of degree of engagement with or by therapists? In this study, therapists were instructed to spend no more than 15 minutes per participant in writing responses to emails (ie, maximum 180 minutes per participant over 12 weeks). While some participants did not use the opportunity to write to their therapist, others wrote multiple emails with copious details. Investigating the impact of markers of engagement (eg, number of emails sent to therapist, number of words written in emails) could provide greater insights into mechanisms of change. Alternatively, examining therapeutic alliance and time spent on the modules and degree of homework adherence [67] could also inform about treatment processes. As our previous examination of an automated version of this iCBT indicated medium effect sizes in an uncontrolled naturalistic study [68], factors related to therapist assistance are likely to be important in facilitating greater efficacy in online programs. A following paper will examine some of these issues.

Given the evidence for the efficacy of this intervention alongside other RCTs using iCBT for OCD, we believe that such interventions hold promise as a routine treatment for individuals with OCD, particularly those with similar symptoms as in this study (eg, a majority with mild-moderate symptoms). For example, Andrews and Williams [69] note that 19 out of 20 individuals in their clinic, when given the option, chose iCBT over traditional CBT, meaning the iCBT became the standard treatment. This would be particularly the case for disorders such as OCD, where knowledge of treatment in the health care community has been low [22] and where availability of treatment is consequently limited [24,25]. The flexibility of the protocol means that various treatment constellations could be used. For example, our treatment center (which is subsidized by the Australian Government Department of Health and Ageing) currently offers a free automatic version of the therapy as well as a low-cost therapist-assisted version. Alternatively, community-based clinicians could be trained to provide motivational support while prescribing online training modules as the main component of treatment and then providing limited or brief face-to-face or telehealth support where required (eg, if patients experience difficulties in implementing ERP). This could minimize costs and maximize the advantages of internet-based treatment irrespective of funding models for mental health services. Alternatively, online programs can be used within a stepped care framework, which has been found to be effective in the treatment of anxiety [70].

### Limitations and Strengths

A number of limitations have already been discussed throughout the paper. These include the lack of an inactive control arm, high rate of dropout at posttreatment assessment, and available data being biased toward older participants. Nonetheless, based on the evaluation framework from Öst [47], our study has several strengths. Broad recruitment strategies were used and participants were only excluded if they met primary criteria for other major disorders or reported current active suicidal ideation; thus, we are confident that the sample constituted a good representation of patients seeking online treatment for OCD. Furthermore, the use of well-trained blind evaluators, structured interviews to establish initial clinical diagnosis, and psychometrically sound outcome measures added to the quality of our study. Although the clinician contact was limited to 1 email per week for each participant, all therapists were trained using an online training module for e-therapists and supervised biweekly by an experienced clinical psychologist trained in e-therapy who provided supervision of e-therapists ensuring ongoing checks for treatment adherence and therapist competence. Equality of treatments, bone fide nature of the control condition, randomization to treatment condition, equality of maximum therapist engagement, and control of concomitant treatments were all strengths of this RCT. Although ITT analyses were used to control for this, participant attrition from data collection was a distinct limitation and may have impacted effect sizes.

### Conclusions

Overall, this paper established the large magnitude effect of a 12-module therapist-assisted iCBT program for OCD and the moderate magnitude effect of iPRT when embedded within the framework of coping with anxiety in OCD-relevant situations. The iCBT program was significantly more efficacious than iPRT, and the sequential addition of iCBT immediately following iPRT was no more efficacious than iCBT alone. Recovery rates were not as high as those reported in previous literature, although participant characteristics and recruitment strategies may account partially for these findings. While only pretreatment OCD symptom severity predicted outcome, younger people were more likely to drop out before assessment. Nonetheless, the study supported iCBT as a useful form of treatment for OCD, a disorder characterized by shame, delayed help-seeking, and poor access to expert treatment. The integration of digitally delivered treatment options into health systems and policies therefore seems an important development in managing the mental health challenges of communities.

## Abbreviations

CONSORT: Consolidated Standards of Reporting Trials
DSM-IV-TR: Diagnostic and Statistical Manual of Mental Disorders, 4th Edition, Text Revision
ERP: exposure and response prevention
HAM-A: Hamilton Anxiety Rating Scale
HAM-D: Hamilton Depression Rating Scale
iCBT: internet-based cognitive behavioral therapy
iPRT: internet-based progressive relaxation training
ICC: intraclass correlation coefficient
ITT: intention-to-treat analysis
MCAR: missing completely at random
NHMRC: National Health and Medical Research Council
OCD: obsessive-compulsive disorder
RCT: randomized controlled trial
SCID-CV: Structured Clinical Interview for DSM-IV Axis 1 Disorders, Clinician Version
YBOCS: Yale-Brown Obsessive-Compulsive Scale

## Appendix

Multimedia Appendix 1 CONSORT‐EHEALTH checklist (V 1.6.1).
